# Case management for the treatment of patients with major depression in general practices – rationale, design and conduct of a cluster randomized controlled trial – PRoMPT (Primary care Monitoring for depressive Patient's Trial) [ISRCTN66386086] – Study protocol

**DOI:** 10.1186/1471-2458-5-101

**Published:** 2005-10-05

**Authors:** Jochen Gensichen, Marion Torge, Monika Peitz, Heike Wendt-Hermainski, Martin Beyer, Thomas Rosemann, Christian Krauth, Heiner Raspe, Josef B Aldenhoff, Ferdinand M Gerlach

**Affiliations:** 1 Institute for General Practice, Chronic Care and Health Services Research University of Frankfurt, Theodor-Stern-Kai 7, 60590 Frankfurt a. M., Germany; 2 Department of General Practice and Health Services Research, University of Heidelberg, Voβbstr. 2, 69115 Heidelberg, Germany; 3 Department of Epidemiology, Social Medicine and Health System Research, Hannover Medical School, Carl-Neuberg-Str. 1, 30625 Hannover, Germany; 4 Institute for Social Medicine, University of Lübeck, Beckergrube 43-47, 23552 Lübeck, Germany; 5 Center for Integrative Psychiatry – University Hospital Schleswig-Holstein – Campus Kiel, Niemannsweg 147, 24105 Kiel, Germany

## Abstract

**Background:**

Depression is a disorder with high prevalence in primary health care and a significant burden of illness. The delivery of health care for depression, as well as other chronic illnesses, has been criticized for several reasons and new strategies to address the needs of these illnesses have been advocated. Case management is a patient-centered approach which has shown efficacy in the treatment of depression in highly organized Health Maintenance Organization (HMO) settings and which might also be effective in other, less structured settings.

**Methods/Design:**

PRoMPT (PRimary care Monitoring for depressive Patients Trial) is a cluster randomised controlled trial with General Practice (GP) as the unit of randomisation. The aim of the study is to evaluate a GP applied case-management for patients with major depressive disorder. 70 GPs were randomised either to intervention group or to control group with the control group delivering usual care. Each GP will include 10 patients suffering from major depressive disorder according to the DSM-IV criteria. The intervention group will receive treatment based on standardized guidelines and monthly telephone monitoring from a trained practice nurse. The nurse investigates the patient's status concerning the MDD criteria, his adherence to GPs prescriptions, possible side effects of medication, and treatment goal attainment. The control group receives usual care – including recommended guidelines. Main outcome measure is the cumulative score of the section depressive disorders (PHQ-9) from the German version of the Prime MD Patient Health Questionnaire (PHQ-D). Secondary outcome measures are the Beck-Depression-Inventory, self-reported adherence (adapted from Moriskey) and the SF-36. In addition, data are collected about patients' satisfaction (EUROPEP-tool), medication, health care utilization, comorbidity, suicide attempts and days out of work.

The study comprises three assessment times: baseline (T0) , follow-up after 6 months (T1) and follow-up after 12 months (T2).

**Discussion:**

Depression is now recognized as a disorder with a high prevalence in primary care but with insufficient treatment response. Case management seems to be a promising intervention which has the potential to bridge the gap of the usually time-limited and fragmented provision of care. Case management has been proven to be effective in several studies but its application in the private general medical practice setting remains unclear.

## Background

Depression is a significant burden of illness [1,2]. Most depressed patients are diagnosed and treated by general practitioners [3,4]. Depression is the third most common reason for a primary care consultation [5]. Patients with depression account for 50% higher health care costs patients than patients who are not depressed [6]. When improving primary health care for chronic conditions, a number of problems have to be resolved: discontinuity and fragmentation of the process of care, lack of co-ordination between different providers, and "the tyranny of urgency" [7]. In the management of depression, these deficits lead to frequent interruption or premature termination of drug therapy, which increases the risk of unfavourable depression outcomes [8,9]. Patient-centred approaches with a focus on empowerment and self management have been recommended [10,7,11]. Case Management may be one approach to improve depression care. Case Management has been defined as "taking responsibility for following-up patients; determining whether patients were continuing the prescribed treatment as intended; assessing whether depressive symptoms were improving; and taking action when patients were not adhering to guideline based treatment or when they were not showing expected improvement" [12]. Case Management consists of five essential components: (1) identification of patients in need of services, (2) assessment of individual patient needs, (3) developing a treatment plan, (4) coordination of care, and (5) monitoring outcomes and altering care when favourable outcomes are not achieved [13].

Reviews of controlled trials including our meta-analysis concluded that Case Management improves patient outcomes – with a moderate effect [12,14,15]. However, most of the studies available were conducted in highly organized Health Maintenance Organizations (HMO) setting using central based stuff to run the intervention. However, it still remains unclear what the effects in a peripheral setting as a private general practice based case management for patients with depression will be like.

## Methods/Design

### Aim of the study

The study examines the efficacy of a GP based case management intervention for patients with depression.

### Scientific hypothesis

Case management (intervention) leads to greater reductions in depressive symptoms than usual care (control group). We hypothesize further that case management leads to a greater increase in adherence (medication) and quality of life.

### Study design

The study is a (prospective) cluster-randomized two-armed intervention study with the practices being clusters. The design of a cluster randomized study was chosen because with this type of study internal validity (absence of confounders) can be optimized, contamination of interventions associated with patient randomization is not possible.

### Sample size

Sample size calculations for cluster randomized trials differ completely from sample size calculations for common RCTs [16,17]. Based on the main outcome parameter depression symptom and the main outcome-assessment instrument (GERMAN-PHQ) [18] we performed a power calculation with the method of Hayes and Bennett [19]. It is provided to record a clinical relevant intervention effect of the Case Management from yet 10% up to the primary size, thus an improvement of the depression score (PHQ-D). Assuming an effect of 35 % (PHQ – Score difference from 18,6 to 12.9) in intervention group and a effect of 25 % in control [20] (PHQ Score – 18.6 to 12.9) we have to detect a minimal difference of 1,86. With an alpha of 5%, a beta of 20%, a SD of 6.1 and a ICC 0.1 we are calculating N = 680 (drop-out 30% included). Every arm of the study (34 practices) has to include 10 Patients.

### Recruitment of GPs and randomization

**Figure 1:**
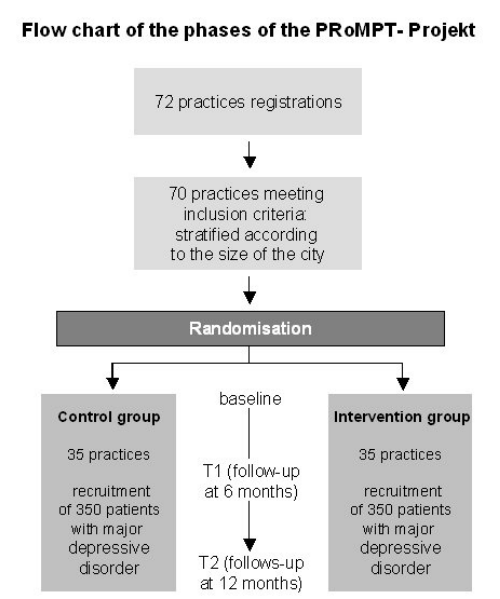


As described above, the GPs form the unit of randomization (cluster). It was decided to address and to include only those practices that have a contract with all German insurances, because 90 % of care provision is covered by this type of practices. About 1600 GPs in the city of Frankfurt and the region near by (federal state of Hessen) were informed by mail about the study and invited to a meeting. After this meeting, 72 GPs gave their written consent to participate in the study. Based on detailed information about the practice and the GP, the inclusion criteria were checked (no exclusive clinical specialization) and two practices had to be excluded due to the inclusion criteria. The 70 GPs were stratified according to the size of the city where the practice is located and then randomized to the intervention group or the control group. The procedure was done by an independent assistant who is not familiar to one of the participating doctors and is not a person of the project team. A randomization protocol was written. (Figure 1)

### Case manager inclusion criteria

For the practice nurses who wanted to engage in the study, the following criteria are recommended: formal training as practice nurse and at least one year work experience after completion of formal training, and participation in the study case management work shops.

### Patient inclusion criteria

Adult Patients, diagnosed with a Major Depression Episode, aged from 18 to 80 years, capability to give informed consent, sufficient knowledge of the German language and indication for antidepressive treatment (medication and/or psychotherapy) can be included. Diagnostic procedure consists in self-report of depressive symptoms via PHQ-9 [18] and subsequent validation in a clinical interview. Participating practices keep an alphabetic record of their patients who are already on treatment. Five Patients from this list are contacted in consecutive order of appearance in the practice and informed about the option to participate in the study. After checking the inclusion criteria and receiving the informed consent, patients receive the questionnaires. Afterwards, five new patients, fulfilling the same criteria, are to be recruited on a screening-day.

### Data collection

After giving their informed and written consent to participate in the study patients will receive the questionnaires. The completed questionnaires will be turned back to the practice where there a collected from a member of the project team when the practice has recruited 10 patients. The envelopes are opened at the university and scanned ("eyes and hands ™ FORMS "-Software, Version 5-2 of Read Soft). A TIF-file is generated out of each questionnaire to avoid any data-manipulation and to have a medium for data storage. The scanned data are transferred into the SPSS files. For documentation and data reporting in publications CONSORT recommendations for cluster randomized trials are considered [21].

### Outcome-parameter

Table 1 displays the outcome parameters and additionally used instruments. The primary outcome is the depression score assessed by the PHQ-9 questionnaire. The PHQ is an internationally applied instrument for the screening in primary care of the major mental disorders. The German version is also validated [18]. Secondary outcomes include:

**Table 1:** Outcomes and instruments

**Outcome-Parameter (Patient)**	**Instrument**
**Primary Outcome**	
Depression	PHQ-9
**Secondary outcome**	
Depression	BDI (Beck-Depression-Inventar)
Quality of life	SF-36
Adherence to medication	Adapted version of Moriskey adherence questionnaire
Health care utilization	questionnaire, retrospective chart review
Patient satisfaction	Modified EUROPEP
Suicide attempts	questionnaire
Days out of work	questionnaire

• the Beck-Depression-Inventory (BDI) [22]

• self-reported adherence (adapted from Moriskey) [23]

• Quality of life (SF-36) [24]

• Health Care utilization (referrals to specialists, days in clinic); data retrieved form patients chart

• Patient satisfaction (modified EUROPEP-questionnaire) [25]

• Suicide attempts

• Days out of work

These data will be compiled from patient questionnaires and patients chart review. All instruments are well validated and frequently used in international studies. Assessments are done three times: baseline (T0), follow-up after 6 months (T1) and follow up after 12 months (T2).

### Intervention

The practice nurses of the intervention group will be trained to case manager in two work-shops with the following contents: main features of the disorder, communication skills, telephone monitoring and documentation. A follow-up work-shop is scheduled to supervise the telephone-monitoring, and each practice is contacted bimonthly from the project team to advice concerning assessment, documentation and intervention.

The intervention comprises the following aspects:

1. The case manager begins with an introduction session which aims at establishing contact, explaining the patient his/her function, delivering information about the disorder and the self-management tools (education). GPs will also receive a written patient leaflet which provides information about the cause and the treatment possibilities as well as coping strategies and a list of self help books.

2. The case manager contacts the patient for telephone monitoring (10–15 minutes). Based on a structured interview, the case manager will ask the patient first two-weekly (the first two months) and than in intervals of 4 weeks about the status of depression, adherence, side effects of medication and goal-attainment (Depression Monitoring List – DeMol, Gensichen unpublished)

3. After each patient contact, a short report will be given to the family doctor.

In both the intervention and the control group, GPs will receive a summary of evidence based treatments of depression in a primary care setting and these information contain amongst others, the NHG guidelines. There is no implementation strategy in the control group.

### Timeframe of the study

The study team has already randomized the 70 GPs who have declared their willingness to participate in the study and who accepted random assignment to the different groups. The study protocol was approved by the ethics commission of the University of Frankfurt (Approval-Nr. E 26/05).

The patient enrollment has already started and up to now, about 220 patient are included who completed baseline assessment.

### Description of risks

To our knowledge, serious risks or undesired effects of completing questionnaires are not reported in the literature. There are no specific risks related to the study.

### Ethical principles

The study is planned and conducted in accordance with medical professional codex and the Helsinki Declaration of 1996 as well as the German Federal Data Security Law (BDSG).

Patients participate in the study voluntarily. They are informed that they can cancel at any time their participation without disclosing reasons for their cancellation and without negative consequences to their future medical care.

### Patient informed consent

Patients receive written and spoken information about the main features of the study; i.e. about potential benefits for their health and potential risks prior to their consent and participation in the study. In case of acceptance, they sign the informed consent sheet.

In case of study discontinuation data will be extinguished, except the patient affirms explicitly the further analysis of his data.

### Vote of the ethics committee

The study protocol was approved by the ethics committee of the University of Frankfurt previous to the start of the study in April 25, 2005. Inclusion of patients/participants did not start unless there was a written and unrestricted positive vote of the ethics committee. This vote was received in March 2005.

### Data security/disclosure of original documents

The patient names and other confidential information are secured by the medical confidentiality rules and are treated according to German Federal Data Security Law (BDSG). The results of the patient questionnaires are not accessible to the GPs.

All study related data and documents are stored on a protected central server of the Frankfurt University Clinic. Only members of the study team have access to the respective files.

Intermediate and final reports are stored in the office of the Department of General Practice and Health Services Research at the Frankfurt University Clinic.

## Competing interests

The author(s) declare that they have no competing interests.

## Authors' contributions

JG, MT, MP and MB conceived and performed the study and draft manuscript. HWH and HR developed the data-management. CK performed health economical aspects TR, JA and FMG participated in the study design. All authors read and approved the final manuscript.

## Pre-publication history

The pre-publication history for this paper can be accessed here:


